# Sea butterflies in a pickle: reliable biomarkers and seasonal sensitivity of *Limacina retroversa* to ocean acidification in the Gulf of Maine

**DOI:** 10.1093/conphys/coae040

**Published:** 2024-06-21

**Authors:** Amy E Maas, Gareth L Lawson, Alexander J Bergan, Zhaohui Aleck Wang, Ann M Tarrant

**Affiliations:** Bermuda Institute of Ocean Sciences, School of Ocean Futures, Arizona State University, 17 Biological Station, St. George’s GE01, Bermuda; Biology Department, Woods Hole Oceanographic Institution, 266 Woods Hole Road, Woods Hole, MA 02543, USA; Biology Department, Woods Hole Oceanographic Institution, 266 Woods Hole Road, Woods Hole, MA 02543, USA; Conservation Law Foundation, 62 Summer St, Boston, MA 02110, USA; Biology Department, Woods Hole Oceanographic Institution, 266 Woods Hole Road, Woods Hole, MA 02543, USA; Marine Chemistry and Geochemistry Department, Woods Hole Oceanographic Institution, 266 Woods Hole Road, Woods Hole, MA 02543, USA; Biology Department, Woods Hole Oceanographic Institution, 266 Woods Hole Road, Woods Hole, MA 02543, USA

**Keywords:** Calcification, gene expression, respiration, thecosome

## Abstract

The passive dissolution of anthropogenically produced CO_2_ into the ocean system is reducing ocean pH and changing a suite of chemical equilibria, with negative consequences for some marine organisms, in particular those that bear calcium carbonate shells. Although our monitoring of these chemical changes has improved, we have not developed effective tools to translate observations, which are typically of the pH and carbonate saturation state, into ecologically relevant predictions of biological risks. One potential solution is to develop bioindicators: biological variables with a clear relationship to environmental risk factors that can be used for assessment and management. Thecosomatous pteropods are a group of pelagic shelled marine gastropods, whose biological responses to CO_2_ have been suggested as potential bioindicators of ocean acidification owing to their sensitivity to acidification in both the laboratory and the natural environment. Using five CO_2_ exposure experiments, occurring across four seasons and running for up to 15 days, we describe a consistent relationship between saturation state, shell transparency and duration of exposure, as well as identify a suite of genes that could be used for biological monitoring with further study. We clarify variations in thecosome responses due to seasonality, resolving prior uncertainties and demonstrating the range of their phenotypic plasticity. These biomarkers of acidification stress can be implemented into ecosystem models and monitoring programmes in regions where pteropods are found, whilst the approach will serve as an example for other regions on how to bridge the gap between point-based chemical monitoring and biologically relevant assessments of ecosystem health.

## Teaser

Despite seasonal variability, pteropods exposed to acidification over multiple seasons reveal consistent patterns in gene expression and shell condition that can be used as bioindicators of ocean acidification stress.

## Introduction

Anthropogenic activity since the industrial revolution has released a large amount of carbon dioxide (CO_2_) into the atmosphere (~2400 GtCO_2_)(Friedlingstein *et al.,* 2022; IPCC, 2022), a substantial fraction of which (~30%) has been dissolved in ocean waters (Gruber *et al.,* 2019). The addition of this excess CO_2_ into marine systems profoundly modifies the acid–base chemistry of seawater, shifting equilibrium towards a lower pH and a lower saturation state for calcium carbonate compounds (Doney *et al.,* 2009). This process, called ocean acidification (OA), tends to dissolve the skeletal components of marine organisms made from calcium carbonate, and has been shown to impact rates of growth, calcification, gene expression, survival and development for a range of species (Kroeker *et al.,* 2010; Espinel-Velasco *et al.,* 2018), although sensitivity is taxonomically heterogeneous, often relating to the presence of a shell, it is additionally moderated by ecological, physiological and ontogenetic factors (Hancock *et al.,* 2020; Leung *et al.,* 2022).

As our awareness of the repercussions of acidification has risen, there have been concerted efforts to increase monitoring of the carbonate chemistry and acidification of both open-ocean and coastal systems (Wang *et al.,* 2013; Bates *et al.,* 2014; Tilbrook *et al.,* 2019). The objective of monitoring programmes is to synthesize observations of chemistry and biology into information relevant for policy development and resource management (McLaughlin *et al.,* 2015; Cross *et al.,* 2019; Doney *et al.,* 2020). Although there has been progress in implementing observations of chemistry into regional predictive assessments of ecosystem risk (Cooley *et al.,* 2015; Hare *et al.,* 2016), translating laboratory observations of organismal OA sensitivity to *in situ* impacts has been difficult to accomplish (Weisberg *et al.,* 2016; Doo *et al.,* 2020). Plasticity in species responses, as well as seasonal or regional variability of exposure, parental provisioning, ontogeny and other environmental and biological factors, play large roles in our current uncertainty of population-level effects of OA.

Bioindicators are biological variables with a clear relationship to environmental risk factors that can be applied to implement ecologically relevant water quality criteria and to model thresholds for assessment and management (Weisberg *et al.,* 2016; Bednarsek *et al.,* 2019). One common example is the abundance of coliform bacteria (the biological indicator) as a proxy for faecal contamination in water (the environmental stressor). In the case of OA, in order for a bioindicator to be useful, it would need to correlate strongly with the chemical measures of acidification (i.e. pH), or the saturation state of calcium carbonate compounds used for calcification. Development of indicators that can serve as a proxy for these chemical measures hold promise for improved OA monitoring, as these phenotypic presentations of health are typically based on responses integrating over longer timescales and broader spatial scales than the point-based chemical measurements, whilst also being directly linked to the metrics stakeholders care about—the health of organisms and ecosystems.

The aragonite-shelled euthecosomatous pteropods have emerged as a potentially viable source of phenotypic bioindicators of ecosystem acidification stress. These organisms are often referred to as thecosome pteropods or just pteropods in the literature, and are commonly referred to as sea butterflies. The respiration rate, gene expression and shell condition of these organisms are influenced by OA in the laboratory (Manno *et al.,* 2017; Maas *et al.,* 2018; Bednarsek *et al.,* 2019), and there is strong evidence that their shell condition can be an indicator of OA exposure in modern oceanic conditions (Bednaršek *et al.,* 2012; Bednarsek and Ohman, 2015; Mekkes *et al.,* 2021; Niemi *et al.,* 2021; Bednaršek *et al.,* 2022). Pteropod shells are made of a more soluble calcium carbonate compound (aragonite) than the form used by most planktonic species. These shells are impacted at a higher saturation state (ΩAr = 1.5) than would be predicted from pure chemical equilibrium (ΩAr = 1.0), the point at which aragonite is predicted to dissolve (Bednarsek *et al.,* 2019). This suggests that their shell condition could serve as an ‘early warning’ of OA impacts for other more commercially and ecologically important shelled species. Natural phenology and seasonal cycles have, however, been demonstrated to influence phenotypic responses of thecosomes to OA (León *et al.,* 2020; Maas *et al.,* 2020), confounding and potentially obscuring the correlations between saturation state and the gene expression, respiration and shell condition of these potential bioindicators. A meta-analysis of the response metrics was only able to find consensus on thresholds of response (Bednarsek *et al.,* 2019), rather than develop consistent predictive relationships between saturation state and a response variable.

Previously proposed pteropod-based bioindicators that relate to saturation state include shell dissolution (using standard error of the mean (SEM)), shell calcification (using calcein staining) and survival (Bednaršek *et al.,* 2017). Both calcification and survival are only valuable for the detection of tipping points, as they require the capture and maintenance of organisms for a period of time and cannot be used with wild-caught animals to assess their prior exposure. Shell dissolution using SEM evaluation, by contrast, can be applied to animals collected directly from the wild. Studies have shown that SEM evaluation, which is qualitative rather than quantitative, is not a sensitive metric across the full range of saturation states experienced by thecosomes, and results are not highly repeatable between users (Oakes *et al.,* 2019). Furthermore, there is an extensive debate in the literature as to the effects of animal handling, associated with the lab studies that use the SEM method (Bednarsek *et al.,* 2016; Peck *et al.,* 2016a; Peck *et al.,* 2016b; Miller *et al.,* 2023), making it a less desirable approach to widely implement in monitoring programmes. SEM is only one of the shell quality metrics that have been implemented, with various other analyses, including transparency, opacity, and a Limacina Dissolution Index also used throughout the literature (reviewed in Oakes *et al.,* 2019). Although the direct mechanism by which saturation state causes changes in these shell condition metrics has not been identified, these approaches appear to document changes in the interaction between the aragonite crystals and the protein matrix that make up the shell. The arrangement of the crystal structure seems to be affected, causing a ‘frosted’ white appearance in oblique light, and a brownish colouration with transverse lighting. Of these, the quantitative microscopy techniques (dry shell opacity and transparency) have been revealed to have both high repeatability and sensitivity across a large range of saturation states (Bergan, 2017; Oakes *et al.,* 2019). Quantitative relationships between duration and severity of exposure, or analyses of seasonal or ontogenetic differences in CO_2_ sensitivity are still missing.

Beyond shell condition, thecosome respiration rate and gene expression have been suggested as potential bioindicators for OA. Respiration rate response to acidification has been shown to be variable and dependent on the presence of co-stressors (Comeau *et al.,* 2010; Lischka and Riebesell, 2012; Seibel *et al.,* 2012), although the cause of this variability is unclear. Gene expression patterns suggest similar suites of upregulated and downregulated genes are present during periods of high CO_2_ exposure across species and studies (Johnson and Hofmann, 2017; Maas *et al.,* 2018; Maas *et al.,* 2020). They are overlaid by patterns of seasonal gene expression that could be either signals of environmental exposure or ontogenetic and developmental phenology. Consequently, thus far it has been difficult to isolate distinct phenotypes that are associated with *in situ* exposure to CO_2_. To precisely identify biomarkers, we thus require studies that disentangle seasonal responses from exposure level responses, isolating natural variability from CO_2_-specific responses. The best experimental design is thus a repeated controlled laboratory experiment embedded within natural seasonal variability.

**Figure 1:**
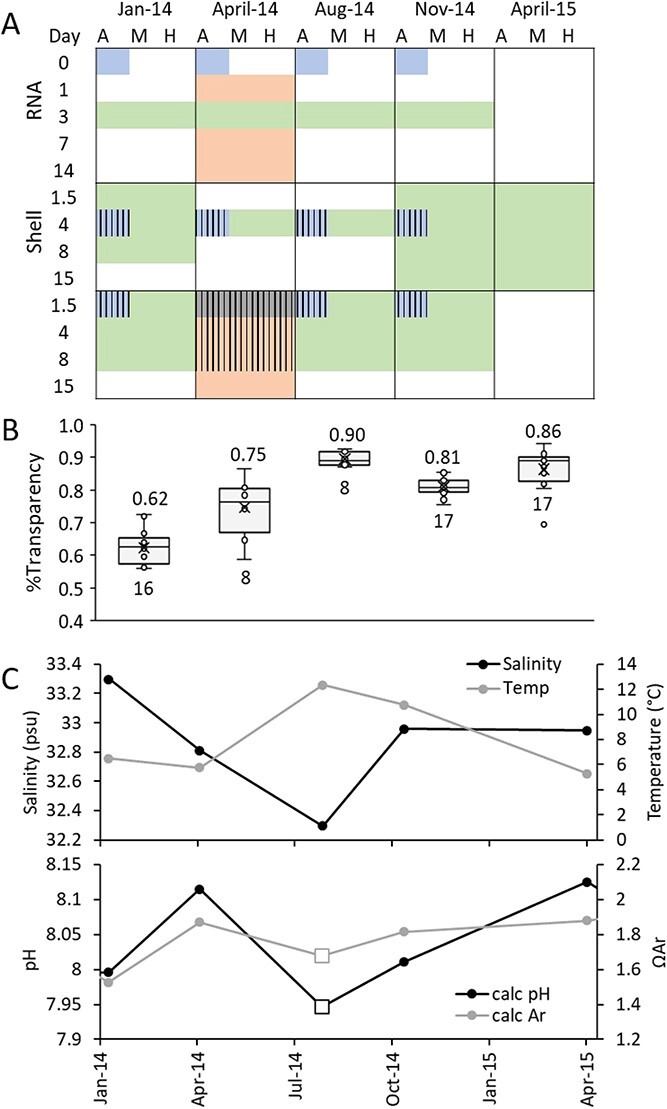
**Sampling design and seasonal profiles.** The sampling plan (A) consisted of taking animals from five different cruises and exposing them for up to 15 days to three CO_2_ treatment levels: ambient (A), medium (M) and high (H). Detailed carbonate chemistry from these exposures is available in Table 1. Some data from these experiments has previously been published in a durational study (Maas *et al.,* 2018; peach), and an *in situ* seasonality study (Maas *et al.,* 2020; blue), but the majority of the samples described herein were analysed explicitly for this project (green). Samples from a prior analysis that were also used in this analysis are demarcated by stripes. (B) Shell transparency from animals maintained in ambient conditions for 0–4 days, representing *in situ* shell condition. Average value is denoted above, and number of biological replicates below the data. (C) Upper water column hydrographic (CTD; average 0–60 m) and carbonate chemistry bottle sampling (bottle sampling; average 0–60 m, *n* = 3) demonstrates the seasonal variability in environmental conditions that are associated with phenological patterns of growth and reproduction, with a pronounced spring spawning event peaking around May and a smaller late fall reproductive event as described in Maas *et al.* (2020).

*Limacina retroversa* is a dominant euthecosome pteropod species in the temperate North Atlantic, broadly distributed in coastal and open-ocean waters (Bé and Gilmer, 1977). Although pteropods, with their delicate shells and mucous-web feeding, are notoriously difficult to culture (Howes *et al.,* 2014), substantial progress has been made in rearing *L. retroversa* in aquaria, making it a strong candidate as a model pteropod species (Thabet *et al.,* 2015; Bergan *et al.,* 2017; Maas *et al.,* 2018). This study assessed seasonal patterns of physiological responses of *L. retroversa*, to laboratory exposure to CO_2_. These organisms are found throughout the year in the Gulf of Maine region where they experience natural seasonal cycles in saturation state (Wang *et al.,* 2017; Maas *et al.,* 2020). Prior work in the area has documented pulses of reproduction in the spring and fall, reduced shell transparency in the winter when environmental CO_2_ is naturally elevated, increased respiration rate during the spring bloom and seasonally distinct patterns of gene expression (Maas *et al.,* 2020). Additionally, laboratory experiments conducted exclusively during the spring bloom have demonstrated that there are increases in respiration rate, reduced shell transparency and increasing numbers of differentially expressed genes with increasing intensity and duration of CO_2_ exposure (Maas *et al.,* 2018). Here we use four seasonally repeated laboratory exposures to CO_2_ during 2014 to determine whether there is plasticity in such responses in relation to ontogenetic and environmental variation, whilst identifying consistent biological markers of acidification stress that would be appropriate for implementation into biomonitoring projects (Fig. 1). A fifth experiment in spring of 2015 was additionally conducted to expand shell response data.

## Materials and Methods

*Limacina retroversa* were collected at five different times from the Gulf of Maine, and held in three CO_2_ treatments for up to 15 days of exposure. They were sampled for respiration rate, shell condition and gene expression at various periods throughout their exposure (Fig. 1). The hydrography of the seasonal sampling, as well as the general abundance and vertical distribution of the organisms during these seasonal cruises, has been reported previously (Wang *et al.,* 2017; Maas *et al.,* 2020), providing the ecological context of the seasonality.

### Seawater and animal sampling

Adult *L. retroversa* were collected for physiology experiments during short (1- to 3-day duration) seasonal cruises conducted within the Gulf of Maine (42° 22.1′—42° 0.0’ N and 69° 42.6′—70° 15.4’ W) beginning 29 January, 25 April, 19 August and 4 November 2014 from aboard the *R/V Tioga*, as described in Maas *et al.* (2018, 2020). There was an additional cruise in 25 April 2015 where animals were retained exclusively for studies of the shell as described in Bergan *et al.* (2017). Prior to animal capture, water for animal maintenance was pumped from ~30 m depth using a submersible pump and filtered using a 63-μm mesh filter. Some of this water was transferred into 1-l jars that were placed in a refrigerator at 8°C to keep it at temperature for animal transport. The rest of the water was stored for transport in large covered plastic bins. On the first day of each cruise this water was transported to an 8°C walk-in environmental chamber at Woods Hole Oceanographic Institution where it was deposited into a holding tank (>400 l) and recirculated past a 1-μm filter throughout the duration of experiments (maximally 2 weeks).

*Limacina retroversa* were sampled using a Reeve net with 333-μm mesh and a large cod end. These were short-duration (<1 h) oblique tows (vertical speed 5–10 m min^−1^, 1- to 2-knot vessel speed) through depth strata where densities had been revealed to be high via prior MOCNESS (Multiple Opening and Closing Net and Environmental Sampling System) and seasonal sampling (generally 80–50 m in the offshore stations and 50–25 m at nearshore sites; described in Maas *et al.,* 2020). Once the net was onboard, the cod end was promptly divided amongst several buckets and diluted. *Limacina retroversa* were sorted from other taxa using a wide-bore plastic pipette and placed at densities between 20 and 40 ind. l^−1^ in the 1-l jars with refrigerated pre-filtered *in situ* water. These jars were stored in coolers or the 8°C refrigerator for transport back to the lab.

### Experimental exposure to CO_2_

As detailed in Maas *et al.* (2018), *in situ* collected pre-filtered water was transferred into three ~100-l pre-equilibration tanks and allowed to bubble for ~12 h prior to the introduction of animals. Gas concentrations were generated with mass flow controllers (Aalborg, Orangeburg, NY, USA) that combined local compressed ambient air (380–440 μatm) and CO_2_ to achieve a medium (~800 ppm) and high (~1200 ppm) treatment (Table 1) as described in White *et al.* (2013). These treatments were chosen to yield supersaturated, near-saturated and undersaturated conditions in each season, with some variability owing to seasonal differences in ambient conditions; these three levels of aragonite saturation state are referred to as CO_2_ treatments, whilst the precise saturation state is referred to as the intensity of the exposure in subsequent analyses. When organisms were brought to the lab after the cruise, this pre-bubbled water was pumped into three 12-l glass experimental carboys per treatment (a total of 9 carboys placed in a semi-randomized pattern in the environmental chamber), where bubbling was continued using micro-bubbling stones.

**Table 1:** Average carbonate chemistry parameters from seasonal laboratory exposures

Sampling period	Treat.	Salinity	Temp (°C)	DIC (μmol kg^−1^)	TA (μmol kg^−1^)	pH	pCO_2_	ΩAr
Jan 2014	A	32	8.02	2101.1	2248.2	7.95	421 ± 14	1.69 ± 0.05
	M	32	8.02	2177.8	2250.0	7.73	728 ± 13	1.08 ± 0.02
	H	32	8.02	2224.6	2257.2	7.59	1016 ± 32	0.81 ± 0.03
April 2014	A	34	8.05	2084.7	2219.0	7.97	471 ± 11	1.54 ± 0.03
	M	34	8.05	2167.0	2223.4	7.74	852 ± 20	0.95 ± 0.02
	H	34	8.05	2202.8	2219.2	7.60	1189 ± 45	0.71 ± 0.02
Aug 2014	A	34	7.77	2039.8	2182.5	8.02	430 ± 14	1.60 ± 0.04
	M	34	7.77	2113.4	2188.3	7.75	718 ± 40	1.07 ± 0.06
	H	34	7.77	2154.0	2189.7	7.64	985 ± 39	0.80 ± 0.03
Nov 2014	A	33	7.57	2084.6	2210.7	8.01	476 ± 17	1.48 ± 0.03
	M	33	7.57	2150.8	2198.8	7.71	864 ± 14	0.88 ± 0.01
	H	33	7.57	2199.7	2202.4	7.58	1310 ± 58	0.63 ± 0.02
April 2015	A	33	8.04	2081.3	2218.5	7.99	449 ± 3	1.58 ± 0.01
	M	33	8.04	2152.3	2221.8	7.78	750 ± 35	1.04 ± 0.04
	H	33	8.04	2202.0	2216.8	7.59	1183 ± 61	0.70 ± 0.04

Dissolved inorganic carbon (DIC), total alkalinity (TA) and pH (total scale) were each measured in all three treatments: ambient, medium and high (A, M, H, respectively; sampling frequency and full carbonate chemistry in Supplementary Table S2). The DIC/TA pairs were used with measured salinity and temperature (Temp) values to calculate pCO_2_ (ppm ± standard error) and the aragonite saturation state (ΩAr ± standard error) using the programme CO2SYS.

Animals were individually distributed randomly into the pre-bubbled experimental carboys at densities of 20–25 individuals l^−1^. In all of the experiments except November 2014, only those individuals that had been collected on the last day of the cruise were used in the experiments. Due to low sampling density in November, individuals were used from the last 2 days of cruise sampling. After placement into the experimental containers, animals were fed a mixture of *Rhodomonas lens* and *Heterocapsa triquetra*, and this feeding regime was repeated once every 2 or 3 days as detailed in Thabet *et al.* (2015) and Maas *et al.* (2018). Water changes were conducted after 1 week of captivity using the remaining water in the holding tank following the protocol of pre-bubbling as mentioned above.

### Carbonate chemistry analyses

The temperature of the environmental chamber was measured continuously throughout the experiment using the temperature sensor associated with the FireSting fibre-optic oxygen meter (PyroScience, Aachen, Germany). Salinity was measured from the experimental carboys using a seawater refractometer (Hanna Instruments, model 96 822) every 2–3 days and during water changes. The pH of each carboy was determined using a USB 4000 (Ocean Optics, Dunedin, FL, USA) spectrometer with an Ls-1 light source and a FIA-Z-SMA-PEEK 100-mm flow cell every 2–3 days using a 2-mM m-Cresol purple indicator dye and as described in Maas *et al.* (2018). Measurements of dissolved inorganic carbon (DIC) and total alkalinity (TA) were conducted on bottle samples collected from pre-bubbled water at the start of the experiment, before and after the water change and the end of the experiment as detailed in Maas *et al.* (2018). Samples were collected in 250-ml Pyrex borosilicate glass bottles, each of which was poisoned with saturated mercuric chloride, following published best practises for seawater CO_2_ measurements (Dickson *et al.,* 2007; Riebesell *et al.,* 2010). DIC was measured using an Apollo SciTech DIC auto-analyser, whilst TA was measured using an Apollo SciTech alkalinity auto-titrator, a Ross combination pH electrode and a pH meter (ORION 3 Star) based on a modified Gran titration method (Wang *et al.,* 2017). Both DIC and TA measurements were calibrated by using Certified Reference Materials (CRMs) provided by Dr A. Dickson at Scripts Institute of Oceanography. The aragonite saturation state (ΩAr) and pCO_2_ during each experimental time point were calculated using concurrently collected DIC-TA pairs, or using the more frequent pH measurements with the TA pair from the closest water change. The seawater carbonate chemistry calculations were made with the CO2SYS software (Pierrot *et al.,* 2006), using constants K1 and K2 by Lueker *et al.* (2000), the KHSO_4_ dissociation constant from Dickson (1990) and the borate relationship from Lee *et al.* (2010).

### Respiration experiments

Respiration measurements were started on Days 1, 3 and 7 of the seasonal experiments, following the protocol detailed in Maas *et al.* (2018). Typically nine individuals were respired per treatment, with final replication ranging from 5 to 9 individuals, and an average number of 8 biological replicates per treatment at each time point and season. Prior to being placed in respiration chambers, individuals were removed from the experimental carboys and placed in a 1-l beaker at densities of 15 ind l^−1^ with 0.2-μm filtered pre-bubbled *in situ* treatment-specific water for 8–12 h to allow for gut clearance. Then they were transferred to custom small-volume glass respiration vials containing fresh 0.2-μm filtered pre-bubbled *in situ* treatment-specific water. Each vial contained an optically sensitive ‘spot’ (OXFOIL: PyroScience, Aachen, Germany) for oxygen sensing. The volume of the chamber was then adjusted to between 2 and 3 ml and closed. A control, filled with water but left without an animal, was set up every fourth chamber for bacterial respiration measurements. The oxygen concentration in each chamber was then measured using a FireSting fibre-optic oxygen meter (PyroScience, Aachen, Germany). The optode was calibrated using air-saturated seawater and zeroed using a 2% sodium sulphite solution at the start of each seasonal experiment. At the conclusion of the respiration experiment (~24 h) the O_2_ concentration was again measured for each chamber. Consequently, animals were sampled after a total exposure duration of 36 h, 4 days and 8 days. The chambers were then weighed wet and emptied and weighed dry to get an accurate estimate of the water volume. Each organism was visually inspected to verify if it was still alive and then was briefly rinsed with DI water, placed in pre-weighed aluminium dish and weighed on a Cahn microbalance (C-33). After weighing, individuals were put in a drying oven at 70°C for >3 days and weighed again. Final oxygen consumption rates were calculated based on the total wet mass and the change in oxygen consumption between the final and initial oxygen measurements (μmol O_2_ g_wm_^−1^ h^−1^). The results were not corrected for the low bacterial respiration from the control chambers that averaged to 0.0002 μmol O_2_ h^−1^, which accounts for <5% of the oxygen consumption in an experiment.

There was some variability in the temperature of the environmental rooms across the experiments. Although the rooms achieved 8.1 ± 0.5°C for most experiments, an equipment failure for the chiller unit during the first day of one cruise (August 2014; 5.6°C) resulted in unexpectedly cold temperatures and the need for a temperature correction across the dataset. The average temperature of the 24-h respiration experiment was used for the original temperature (T_i_), and the adjusted rates (R_f_) were calculated at 8.0°C using a temperature coefficient (Q_10_) of 2 according to the equation: 


(1) \begin{equation*} {\mathrm{R}}_f={\mathrm{R}}_i\ast \left({{\mathrm{Q}}_{10}}^{\left(\frac{8-{\mathrm{T}}_i}{10}\right)}\right) \end{equation*}

where R_i_ is the final oxygen consumption rate measured for each individual. Although Q_10_ is known to be species-specific in pteropods (Seibel *et al.,* 2007), there are no published studies of Q_10_ for *L. retroversa*. This chosen coefficient is mid-range for the published Q_10_ of congeners (1.6–2.3; Ikeda, 2014, Maas *et al.,* 2011). All temperatures experienced by the pteropods were within the normal annual range of their natural environment (Maas *et al.,* 2020), and the total variation in the average temperature amongst the experiments was small (−2.4 to +0.6°C), so slight variations in actual Q_10_ would not substantially influence the calculated temperature-corrected respiration rate.

Statistical analysis involved first testing for an effect of season, CO_2_ treatment and duration of exposure on the log of mass-specific respiration across the full dataset using a General Linear Model (GLM), with log of wet mass as a covariate and using the statistical programme SPSS. Each season was then explored separately to determine the effect of CO_2_ treatment severity and duration of exposure and differences explored using Bonferroni *post hoc* tests. Metabolic rate was additionally analysed based on the intensity of the CO_2_ exposure level.

### Shell transparency analyses

Shell transparency analysis was conducted on individuals from all five of the seasonal cruises after 4 days of exposure. For all of the 2014 cruises, individuals that had been used in the respiration experiments were dried as described above, and then analysed for shell transparency following the methods of Maas *et al.* (2020) and Bergan *et al.* (2017). For the 2015 cruise, animals were removed directly from the experimental treatments for transparency analysis. Additional samples from January 2014, November 2014 and April 2015 were taken from individuals exposed for a duration of 36 h, 4 days, 8 days and 15 days and were used to explore general patterns of response to intensity and duration of CO_2_ exposure. Replication ranged from five to nine individuals per treatment for each time point and season, with an average replication of seven individuals.

To remove body tissue, individuals were placed in 8.25% hypochlorite bleach for 24–48 h, rinsed in DI water and dried. These individuals were then photographed under a stereoscopic light microscope at 25× magnification with a background greyscale value of 255. Images were analysed by thresholding the image to black and white and the apertures, as well as any holes, were manually cropped. The degree of light transmittance was then calculated using a custom MATLAB code with the mean greyscale value (range: 0–255) of the pixels of the shell divided by 255 to get a scale of 0 (black) to 1 (white). Using a GLM in SPSS, we assessed whether there was an effect of season, treatment and the interaction term on the transparency of the pteropod shells with differences explored using Bonferroni *post hoc* tests. We then used a GLM to determine whether there was a significant effect of season on the slope of the relationship between the log saturation state and log transparency for each duration of exposure.

A power equation relating saturation state and shell transparency was generated based on the average of the regressions from the 4-day experiments. To estimate the effect of duration, we applied this power equation to the 15-day shell transparency experiments. A theoretically ‘predicted’ shell transparency was calculated for all shells from the medium and high treatments in these experiments, using this power equation and the seasonal specific constant for the sampling period. The difference between the observed transparency and this ‘predicted’ transparency was then plotted versus the duration of exposure to provide a regression that estimates the effect of duration on shell transparency after accounting for the effect of saturation state.

### Gene expression

After 3 days of exposure, freely swimming pteropods were removed directly from the large carboys and immediately preserved in RNA for later analysis of gene expression. Within each treatment, total RNA was extracted using the E.Z.N.A. Mollusc RNA Kit (Omega Biotek) from 5 to 6 biological replicates, each containing a pool of 5–9 pteropods. Three RNA samples were selected from each season and treatment combination (36 samples total) based on spectral profile and RNA yield, and these were submitted to the University of Rochester Genomics Research Center for sequencing. Libraries were constructed using TruSeq Reagents and then sequenced on four lanes of an Illumina HiSeq 2500 (9 samples per lane) as a High-Output v4 125 bp PE project. The sequencing facility used Trimmomatic (v.0.32; Bolger *et al.,* 2014) to eliminate adapter sequences (2:30:10) and removed low-quality scores using a sliding window (4:20), trimming both trailing and leading sequences (13) and leaving only sequences with a minimum length of 25 for downstream use.

Reads from individual RNA samples were then aligned to the transcriptome that was previously generated and annotated in association with this project (Maas *et al.,* 2018), allowing direct comparisons between studies. This assembly has been demonstrated to be sufficiently complete (BUSCO score C: 90.6% [S:65.1%, D:25.5%], F:8.0%, M:1.4%, n:978) to support the analysis (Simão *et al.,* 2015). Alignment was completed using the pipeline packaged with Trinity (Haas *et al.,* 2013), using Bowtie2 (v.2.2.3; Langmead and Salzberg, 2012), and estimates of abundances were made with RSEM (Li and Dewey, 2011). Read mapping statistics indicate a reasonable alignment rate of 73.8 ± 1.3% (Supplementary Table S1). edgeR analysis of differential expression (DE) was performed with R v.3.0.1 (Robinson *et al.,* 2010). Pairwise comparisons were made within each season between ambient samples and either medium or high samples to explicitly test for the effect of CO_2_ during each season. Genes were defined as DE if the false discovery rate and the *P-*value were both <0.05, and the log_2_-fold change was >2 (corresponding to a 4-fold change in expression).

To explore gross patterns of gene expression amongst days and treatments, TMM gene expressions of all samples and treatments were log-transformed and then a Bray Curtis Similarity Matrix was generated for the data using PRIMER. Samples were plotted as an nMDS with the factors month and treatment to determine significant clustering. To explore the effect of CO_2_, a second nMDS was plotted using only those genes that were differentially expressed in a pairwise comparison. The environmental conditions from each experiment (Table 1) were then correlated with these patterns of gene expression to determine which factors were most predictive of transcriptomic responses. The DE genes were compared with prior analyses of the effect of the intensity and duration of CO_2_ exposure in the lab (Maas *et al.,* 2018) as well as the prior *in situ* seasonal expression patterns (Maas *et al.,* 2020). Finally, the log (x + 1) TMM expression level of each gene was correlated with the saturation state of the sample to explore genes that may be valuable quantitative and consistent biomarkers of environmental exposure level.

## Results

### Carbonate chemistry

During the five experiments, which ran for 15 days each, natural seasonal variability in seawater alkalinity and local CO_2_ levels translated into a range of different pH values and saturation states based on the bubbling of *in situ* field-collected seawater with ambient air (380–440 μatm) and CO_2_ to achieve medium (~800 ppm) and high (~1200 ppm) treatments (Table 1; Supplementary Table S2). Although ambient treatment was always supersaturated (ΩAr = 1.69–1.48), sometimes the medium and high treatments overlapped across seasons. Physiological response variables were thus analysed using both the treatment level (as a nominal factor) when looking for seasonal differences and the saturation state (continuous variable) when exploring the relationship between treatment and response variable.

### Respiration experiments

**Figure 2:**
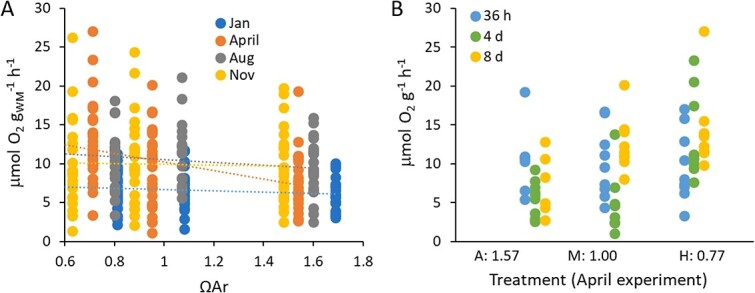
**Measured mass-specific oxygen consumption compared with CO**_**2**_ **treatment levels. A)** Metabolic rate, normalized to total wet mass, for each sampling period (colours) is reported in comparison with the intensity of the CO_2_ exposure as indicated by measured aragonite saturation state. All exposure durations (36 h, 4 days and 8 days) are shown in aggregate as there was no overall effect of duration of treatment on metabolic rate for the full dataset. **B)** When seasons were analysed independently, April was the only period where respiration rate was influenced by CO_2_, with a significant and interactive effect of both the duration (colours) and CO_2_ treatment level (x-axis). Each point represents an individual organismal measurement, with replication ranging from five to nine individuals per treatment at each time point and season.

Respiration rates, as measured after 36 h, 4 days, or 8 days of laboratory CO_2_ exposure during the four 2014 experiments, were significantly influenced by sampling period (F_3, 263_ = 15.374, *P* < 0.001) and duration of exposure (F_2, 263_ = 11.773, *P* < 0.001), whilst treatment was not significant (F_2, 263_ = 2.550, *P* = 0.080; Fig. 2A) in a full factorial analysis. Each sampling period was also independently analysed for the influence of treatment level and duration. The only period where CO_2_ had an influence was April 2014, when level of CO_2_ treatment had a statistically significant effect (F_2, 66_ = 4.948, *P* = 0.010; Fig. 2B), and there was an interactive effect between CO_2_ and duration (F_4, 66_ = 4.886, *P* = 0.002). This was observed as an increasing difference between ambient and the other treatments as duration increased, and was supported by Bonferroni *post hoc* tests, which indicated that the medium and high treatments had a higher respiration rate than ambient after 8 days of exposure. The pattern is suggestive of a transition to an increased metabolic rate when exposed to CO_2_, reached earlier in the high treatment than the medium treatment in April.

### Shell transparency

**Figure 3:**
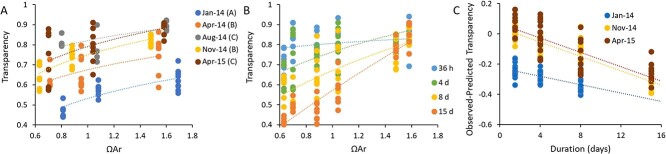
**Effect of season, duration and intensity on shell transparency. A)** There was a consistent effect of the intensity of CO_2_ exposure (as indicated by aragonite saturation state) on shell transparency, where the month of capture (colour; statistical grouping in parentheses) had a significant effect on the intercept (presumably ambient starting transparency; data shown after 4 days exposure, power regression), but no significant effect on the slope of the relationship between transparency and saturation state. **B)** The duration of exposure (colour; time) had an interactive effect with the intensity of CO_2_ exposure, where increasing duration reduced the transparency at increasing intensity (shown excluding the January 2014 data points to provide a similar starting shell condition; power regression). **C)** For the duration experiments a ‘predicted’ shell transparency was calculated based on saturation state (using equation 2), and the difference between the observed transparency and this predicted transparency was plotted versus the period of exposure to determine the effect of duration on shell transparency. Each point represents an individual organismal measurement. Replication ranged from 5 to 9 individuals per treatment at each time point and season.

The shell transparency of organisms was measured for all three treatment levels after 4 days of laboratory exposure in each of the five sampling periods. Shells unexposed to elevated CO_2_ had higher transparency (max = 0.94), whilst those exposed to higher intensity or longer duration CO_2_ exposure had a lower transparency (min = 0.44). Although there was a significant effect of sampling period (GLM; F_4, 97_ = 63.451, *P* < 0.001) and a clear effect of treatment (F_2, 97_ = 44.441, *P* < 0.001), there was no interactive effect between sampling period and treatment (F_8, 97_ = 1.684, *P* = 0.112), meaning that there was no significant difference in the relationship between shell transparency and treatment amongst sampling periods (Fig. 3A). Our earlier work in the Gulf of Maine region has demonstrated lower transparency in shells from field-caught pteropods during the winter, when saturation state is naturally at its lowest point (Maas *et al.,* 2020). Shells from January retained this significantly lower transparency compared to all of the other seasons, whilst those from April 2014 and November had intermediate transparency, and those from August and April 2015 had the highest transparency (Bonferroni *post hoc* analysis; Fig. 3A). Synthesizing this data provides a quantitative relationship between the saturation state and shell transparency that is best expressed as a power function: 


(2) \begin{equation*} Transparency= alpha\left({Saturation\ State}^{0.255}\right) \end{equation*}

where the exponent is calculated as the average exponent from each independent season (*n* = 5; SE = 0.032). The constant (*alpha*) is related to the prior exposure to CO_2_ in the environment, and was strongly correlated (R^2^ = 0.9885) with the previously measured shell transparency of field-caught organisms (Day 0) from the same season (Maas *et al.,* 2020):


(3) \begin{equation*} alpha=1.0293\left( Day\ 0\ Transparency\right)-0.1087 \end{equation*}

Using this equation with the highest observed shell transparence at Day 0, we estimate that alpha = 0.82 for pristine field-caught shells uninfluenced by reduced environmental saturation states (transparency ~0.9).

To assess the combined effects of the intensity and duration of CO_2_ exposure on shell transparency, we conducted additional experiments during January 2014, November 2014 and April 2015 in which we used all three CO_2_ treatment levels, and measured transparency after 36 h, 4 days, 8 days and 15 days. Ambient treatments (ΩAr = 1.69–1.48) retained a similar shell transparency for the duration of the experiment, whilst there was a statistically significant reduction in transparency over time for medium and high treatments, with an increase in the exponent of the relationship between saturation state and shell transparency over the duration of the experiments (Fig. 3B). Although there was a seasonal specific starting transparency that influenced the function, there typically was a statistically similar exponential relationship amongst seasons at each duration (SF1).

Since the effect of treatment did not statistically emerge until after the 36-h treatment (SF1), the effect of duration was modelled by first calculating a predicted Day 4 transparency (D4T) for each observed saturation state in the duration experiments (using Eqn.2 and the observed alpha for each sampling period). The difference between the observed transparency and the D4T value can then be attributed to a change in shell condition caused by the duration of exposure (Fig. 3C). There was no interactive effect between duration and season (F_5, 239_ = 1.897, *P* = 0.096). Knowing that the CO_2_ response is thought to have a threshold at ΩAr ~ 1.5 (Bednarsek *et al.,* 2019), we tested the treatment levels separately. There was no effect of duration on shells from the ambient treatments (SF1; F_3, 76_ = 1.946, *P* = 0.129), whilst there was a similar and significant effect of duration on both the medium (F_3, 75_ = 58.584, *P* < 0.001) and high (F_3, 66_ = 67.140, *P* < 0.001) treatments. Based on our November 2014 and April 2015 datasets we calculated this average linear relationship to be: 


(4) \begin{equation*} Observed\ Transparency-D4T=-0.0238(Duration)+\mathrm{\beta} \end{equation*}

where beta is associated with the starting difference in shell transparency owing to seasonal variation, with an estimated beta of 0.1026 for a shell from a season with no prior exposure to OA. By setting the change in transparency to zero and applying the alpha for a pristine shell, we can calculate that the observed changes in shell transparency emerge after ~2.3 days of exposure (1.7 days prior to the Day 4 measurements).

### Gene expression

**Figure 4:**
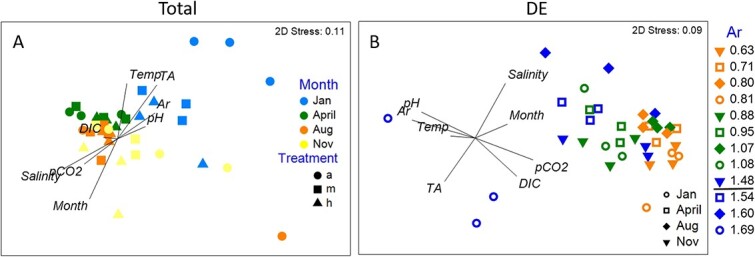
**nMDS of total gene expression (A) and the expression of only those genes that were differentially expressed in one pairwise experimental comparison (B).** Treatments are demarcated as ambient (a), medium (m) or high (h) in the left hand plot and by saturation state in the right hand plot with ambient (blue), medium (green) and high (orange) treatments falling at different levels owing to underlying differences in the carbonate chemistry. The theorized tipping point for saturation state (1.5) is noted in the legend. In the full comparison the experimental condition (Table 1) that was the best predictor of MDS1 (x-axis) was salinity, whilst the best predictor of MDS2 (y-axis) was month. In the analysis using only genes that were DE in one pairwise comparison, the experimental condition that was the best predictor of MDS1 was aragonite saturation state, whilst the best predictor of MDS2 was salinity. In both plots each point corresponds to a biological replicate comprised of a pooled sample of five to nine organisms. There were three resulting biological replicates per treatment per season.

Our prior work on field-caught *L. retroversa* demonstrated seasonal patterns of gene expression indicative of responses to *in situ* variation in saturation state (Maas *et al.,* 2020). Additionally, a 14-day exposure study from April 2014 indicated an effect of both increasing CO_2_ intensity and duration on gene expression, but revealed an interactive effect between CO_2_ treatment and captivity duration (Maas *et al.,* 2018). To minimize captivity effects and allow for a period of physiological response, samples here were analysed in all four experiments from 2014 after 3 days of laboratory exposure to each treatment level. Patterns of total gene expression demonstrate a strong seasonality across the four sampling points studied, with January differing the most from the other three periods (Fig. 4A). When considering only the patterns of expression in transcripts that were statistically differentially expressed (DE) in at least one pairwise comparison between seasons, clustering was more strongly based on treatment (Fig. 4B). In this reduced dataset seasonal clustering was still present (particularly obvious in MD3; Supplementary Fig. S2), emphasizing the strong seasonal influence on CO_2_ responsiveness.

Individuals in the January experiment, which experienced the lowest saturation states *in situ* prior to their capture, had the most pronounced response to elevated CO_2_ exposure (Table 2). The genes identified as DE in January shared the greatest number of DE genes with the previously published analysis of DE genes across seasons in freshly caught individuals in the field (Maas *et al.,* 2020), emphasizing the similarity in response between field and laboratory exposures to CO_2_ (Supplementary Table S3). April organisms were the next most responsive to laboratory exposure, whilst August was the least (Table 2). The largest numbers of shared DE genes were in ambient versus high comparisons in January and April, followed by the medium versus high comparison from November (Supplementary Table S3). Directionality of gene DE was generally conserved amongst studies and seasons (Supplementary Table S3), emphasizing the reliability of these markers.

**Table 2:** Number of differentially expressed genes

Comparison	Jan	Apr	Aug	Nov
A vs H	601	328	21	48
A vs M	300	11	44	7
M vs H	91	89	12	102
Duration	108	176	30	54
Seasonality	361	66	40	49

Ambient (A), medium (M), and high (H), expression profiles were analysed through pairwise comparisons within each month. DE genes from each month were then compared with those that were identified during a companion study of the effect of CO_2_ exposure duration (‘duration’ comparison), conducted during the April cruise and lasting for 14 days (Maas *et al.*, 2018), and the number of shared genes is denoted. Finally, the DE genes from this study were compared with those that were differentially expressed during a seasonal study of *in situ L. retroversa* transcriptome expression (Maas *et al.*, 2020) (‘seasonality’ comparison), and the number of shared genes is also indicated. Annotation of each DE gene, as well as the details of the expression patterns are noted in Supplementary Table S3.

**Figure 5:**
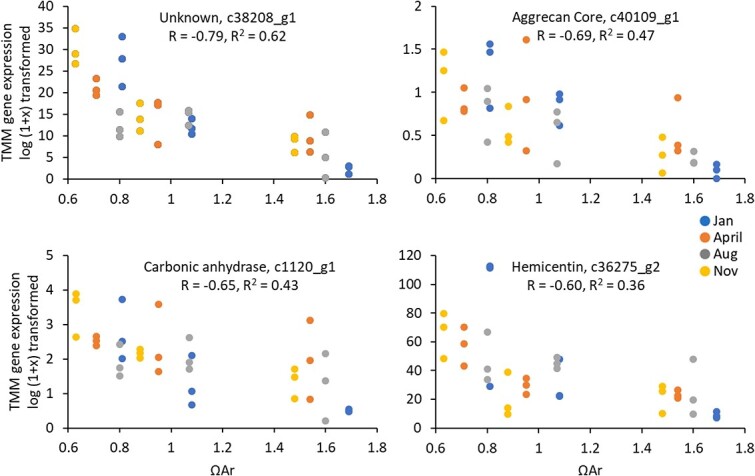
**Effect of season and saturation state on a subset of genes.** There was a relatively consistent effect of the intensity of CO_2_ exposure (as indicated by aragonite saturation state) despite the month of capture (colour) on the gene expression (quantified as the TMM expression after log (1 + x) transformation) for a number of genes after 3 days of exposure. Genes chosen for visual representation were not always the most highly correlated, but represent genes of interest from prior studies and DE analysis. A full list of the R and R^2^ for all DE genes is listed in Supplementary Table S3. In both plots each point corresponds to a biological replicate comprised of a pooled sample of five to nine organisms. There were three resulting biological replicates per treatment per season.

To search for potentially robust biomarkers associated with field and laboratory exposure to CO_2_, the list of genes identified in this study and our companion studies (Maas *et al.,* 2018; Maas *et al.,* 2020) was analysed for shared DE genes (Supplementary Table S3). Subunits of cytochrome c oxidase and NADH dehydrogenase were the most prominent components of the suite of genes typically expressed at higher levels (upregulated) in lower CO_2_ treatments or seasons with higher natural saturation state. Similarly, plasminogen, angiopoietin, fibrinogen, hemicentin, a zinc finger protein, and a number of unidentified sequences were generally expressed at higher levels (upregulated) in higher CO_2_ treatments or seasons with lower natural saturation state. These sequences were frequently annotated by Gene Ontology (GO) terms associated with the extracellular region and calcium ion binding. There were a number of genes (229) whose log (x + 1) transformed TMM gene expression level had a high correlation (abs R > 0.5) with saturation state (Fig. 5; Supplementary Table S3). Expression levels were, however, often variable, and the coefficient of determination, which describes the proportion of variance explained by the correlation, was only high (R^2^ > 0.4) for a small subset of genes (30). The statistical correlation did not track with the frequency of the number of pairwise comparisons that were DE.

## Discussion

Thecosome pteropods have emerged as a group whose phenotypic responses to changes in carbonate chemistry could serve as valuable bioindicators of the severity of ecosystem OA exposure. Prior studies report changes in pteropod shell condition, metabolic rate and gene expression in response to CO_2_ exposure (Bednaršek *et al.,* 2012; Bednarsek and Ohman, 2015; Manno *et al.,* 2017; Maas *et al.,* 2018; Bednarsek *et al.,* 2019; Mekkes *et al.,* 2021). To explore the scope of their seasonal phenotypic plasticity, and to identify consistent biomarkers of CO_2_ stress strongly correlated to saturation state, this study exposed *L. retroversa* to three treatment levels of CO_2_ for a duration of up to 15 days across five study periods spanning all four seasons. Owing to appreciable effects of natural seasonality, respiration was shown to be an unreliable marker for CO_2_ exposure as it was only associated with treatment during periods of high food availability. We identified, however, a suite of genes that may serve as appropriate biomarkers of OA stress after further studies that resolve the effect of duration of exposure. Finally, we demonstrated that shell transparency is consistently influenced by saturation state irrespective of season and, using these findings, we generated regressions of shell condition versus duration and intensity metrics. Together these results substantially advance our ability to quantitatively implement pteropod physiological responses as bioindicators of the severity of ecosystem OA exposure.

The metabolic rate of *L. retroversa* was previously demonstrated to vary seasonally in the wild, presumably in relation to food availability (Maas *et al.,* 2020). This significant difference in metabolism, with higher rates of mass-specific oxygen consumption during the April spring bloom, had an interactive effect with CO_2_ exposure. In the present study, animals were fed identical rations during the experiments, but they would have experienced different food levels prior to collection that presumably affected their energy stores. We observed that metabolic rates were elevated in the more severe treatments (both by intensity and duration) in April and were not affected during other seasons. Our results indicate that higher food availability in conjunction with CO_2_ exposure is associated with an increased energetic expenditure in *L. retroversa* as measured by oxygen consumption. During the rest of the year, the animals appear to be using a basal metabolism, and may have no scope for increased energetic expenditures to apply to CO_2_ response. Similar seasonal variation in sensitivity to CO_2_ has been previously observed in silverside fish larvae (Baumann *et al.,* 2018), where it was potentially attributed to maternal provisioning (Snyder *et al.,* 2018). Food availability, which is related to body stores of energy, has consistently been demonstrated to influence CO_2_ sensitivity (Seibel *et al.,* 2012; Thomsen *et al.,* 2013; Pansch *et al.,* 2014; Brown *et al.,* 2018; Cominassi *et al.,* 2020). Our dataset extends these findings by suggesting that responsiveness to CO_2_ must be inexorably linked with seasonal patterns of productivity and reproduction.

Shell condition, using a suite of different methodologies, has previously been shown to be impacted by CO_2_ gradients in natural populations in a variety of species of the pteropod family Limacinidae (Bednaršek *et al.,* 2012; Bednarsek and Ohman, 2015; Maas *et al.,* 2020; Mekkes *et al.,* 2021; Niemi *et al.,* 2021). Based on these previous *in situ* observations and a number of laboratory experiments, thresholds for changes in shell condition have been identified by meta-analysis near a saturation state of 1.5 (Bednarsek *et al.,* 2019). Variations in species, ontogeny, experimental methodology and treatment levels have, however, made it difficult to define a precise quantitative relationship between saturation state and shell response below that level. We have shown that despite the seasonally different starting levels (Maas *et al.,* 2020), transparency was consistently influenced by the intensity of CO_2_ exposure in the laboratory in all 4-day seasonal experiments, and the threshold of an aragonite saturations state of ~1.5 was statistically upheld. The CO_2_-induced changes to transparency increased over time resulting in an interaction between intensity and duration of exposure, which can arguably be considered to be the total severity of exposure (Bednarsek *et al.,* 2019). Our data demonstrates that changes in shell condition are negligible after 36 h of exposure, but emerge after ~2.3 days. There were very few individuals with transparency observed below the threshold of ~0.4, and these were only present in January in the 8-day high-CO_2_ treatment, suggesting a lower biological limit. By measuring the transparency of a shell directly collected from the environment (T; which is the observed transparency) we can combine the equations 1 and 3 that relate transparency to saturation state, as well as the calculated alpha of 0.81 and beta of −0.0344, to model the range of durations (D), and intensities ($\Omega \mathrm{Ar}$) experienced by the individual using the following equations:


(5) \begin{equation*} \Omega \mathrm{Ar}={10}^{\frac{\log \left(\frac{T+(0.0238D)+0.0344}{0.81}\right)}{0.255}} \end{equation*}

(6) \begin{equation*} D=\frac{T-0.82\ \left({\Omega \mathrm{Ar}}^{0.0255}\right)-0.0555}{-0.0239} \end{equation*}

**Figure 6:**
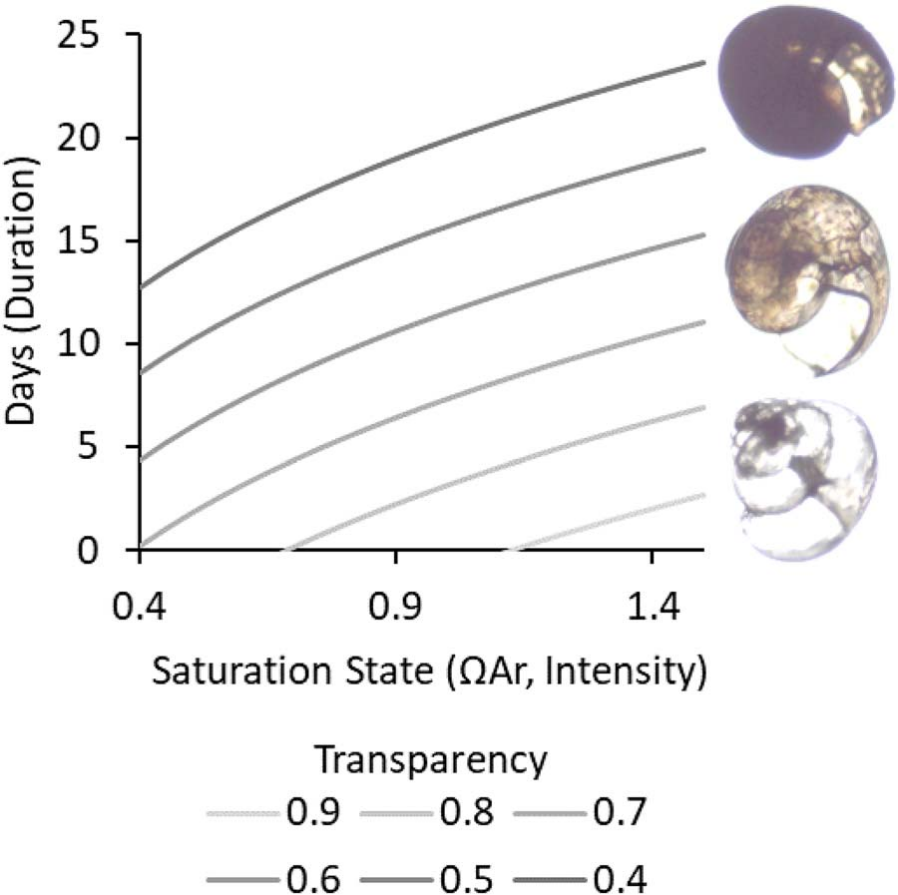
**Potential environmental exposure based on observed shell transparency**. Given a specified shell transparency measured from wild-caught organisms (lines), the ranges of intensity (x-axis) and duration (y-axis) required to produce the observed severity of effect can be calculated between the observed transparency range of 0.9–0.4 and the hypothesized tipping point of ΩAr = 1.5 after >2 days of CO_2_ exposure.

Based on these equations the transparency of shells gathered from the natural environment could provide a sense of the severity of ecological exposure and a range of intensities and durations of CO_2_ exposure organisms experienced in the period prior to capture (Fig. 6). These equations are easy to implement into ecosystem risk models (i.e. Bednaršek*, et al.*, 2019), or ROMS models of saturation state (i.e. Bednaršek *et al.,* 2023) to explore patterns of vulnerability under current and future OA scenarios to direct future conservation efforts. The low cost and simplicity of the measure also makes it attractive for implementation into general ecological monitoring projects. Specifically, shells captured from the environment can be imaged for shell condition to provide characterization of the possible carbonate chemistry history of the region. Although the indicator will never be able to discriminate between intensity and duration of exposure, the prior conditions can be partially constrained by simultaneous point-based or regional continuous or autonomous measurements of carbonate chemistry. This combination of measurements would serve to synthesize an understanding of the accumulated severity of ecosystem exposure to decreased saturation state, and constitute a substantial improvement to our ability to map and monitor OA in pelagic ecosystems.

Prior analyses of gene expression from freshly caught organisms emphasized a strong seasonal pattern in physiology, reflecting annual carbonate chemistry, food availability and ontogenetic/developmental cycles (Wang *et al.,* 2017; Maas *et al.,* 2020). In this study, despite animals being held in similar temperature, carbonate chemistry and food conditions, seasonality was still apparent after 3 days of captivity. This seasonal pattern of gene expression was present even when only considering the genes that were differentially expressed in response to CO_2_. Specifically, organisms from January, the period when the natural environment was measured to have the lowest chlorophyll *a* and lowest saturation state of all sampling points (Maas *et al.,* 2020), had the highest transcriptomic responsiveness to CO_2_. April, the period of highest reproduction and the spring bloom, had the next highest number of DE genes in response to laboratory CO_2_ exposure, although it was mainly measured in the high CO_2_ treatment. These results verify that these animals are actively responding to shifts in CO_2_, and suggest that there is an ontogenetic, nutritional, or prior-exposure dependence. Interpreting this responsiveness in the context of ‘sensitivity’ or fitness is not possible from our data.

Despite the seasonal variability in gene expression response to laboratory CO_2_ exposure, we identified a suite of genes that were frequently at a lower expression in the higher CO_2_ treatments. Those that were identified were associated with energetic metabolism—specifically cytochrome oxidase subunits and NADH dehydrogenase subunits. This is consistent with prior documented transcriptomic responses to high CO_2_ in other thecosome pteropods (Maas *et al.,* 2015; Moya *et al.,* 2016; Maas *et al.,* 2018), urchins (Todgham and Hofmann, 2009; O'Donnell *et al.,* 2010) and coral (Moya *et al.,* 2012). These transcripts may, however, be poor biomarkers of OA exposure owing to the lack of specificity of their response (they have been identified as biomarkers of anoxia and heat stress in other invertebrates (Ma and Haddad, 1997, Mahadav *et al.,* 2009, Woo *et al.,* 2013)). The correlation between the log (x + 1) TMM gene expression levels and the saturation state of the sample was low and the expression levels highly variable for these genes (R = 0.01–0.44; R^2^ < 0.19), which would prevent them from serving as reliable indicators of exposure to acidified water.

In the elevated CO_2_ treatments there were many transcripts with GO terms associated with the extracellular region and calcium ion binding that were upregulated in medium and high treatments. Additionally, specific genes were consistently DE in response to higher CO_2_ including plasminogen, angiopoietin, fibrinogen, hemicentin, a zinc finger protein, and a number of unannotated sequences (Supplementary Table S3). Most of these were also flagged as being DE in the durational study (Maas *et al.,* 2018) and were upregulated in January during the *in situ* seasonal analysis of gene expression (Maas *et al.,* 2020). Some have similarly been identified as being DE in studies of other species of thecosome pteropods (Koh *et al.,* 2015; Moya *et al.,* 2016) and the much more distantly related shelled heteropods (Ramos-Silva *et al.,* 2022). Many of these genes appear, however, to be influenced by seasonality; although the overall pattern of expression was consistent, the precise gene expression level in relation to saturation state was variable, leading to lower correlations and coefficients of determination. This indicates that although these DE genes are consistently effected by OA, there is background variability in their expression levels that are indicative of either pre-exposure in the environment, or seasonal and ontogenetic differences in expression level. Although understanding the processes controlled by these transcripts is important for our understanding of OA, these genes are thus not be ideal biomarkers of pteropod exposure to changing saturation state.

There were, however, 229 genes where the correlation between the log (x + 1) TMM gene expression levels and the saturation state of the sample was relatively high (abs R > 0.5). Fifty-six percent of these were unidentified or uncharacterized in the blast search. GO annotation of the poorly annotated sequences suggests a high number of transcripts associated with the membrane cell component, calcium ion or protein-binding function and the protein glycosylation process. Unlike the DE analyses, these correlations do not account for tipping points in biological response, but are consistent across seasons. These genes likely are good targets for biological monitoring of acidification stress in pteropods, as they are expressed in laboratory exposures in multiple seasons in a consistent relationship to saturation state (Supplementary Table S3). To develop any of these transcripts into an informative biomarker, we would, however, need to conduct a similar multi-season duration analysis as that which was conducted for shell quality (summarized in Fig. 6), since gene expression has previously been demonstrated to be highly responsive to duration of exposure (Maas *et al.,* 2018). These findings would then need to be validated with qPCR approaches to reach a similar level of technical and financial feasibility as shell condition analysis for implementation into routine monitoring. Additionally, the lack of full annotation of many of the most well-correlated and consistently DE transcripts prevents us from having a functional understanding of their role in pteropod biology, pointing to areas of important further research.

Broadly, these findings demonstrate that seasonality plays a large role in the overall gene expression of *L. retroversa* and that there are interactive effects of CO_2_ and seasonality on the respiration rate of this species that appears to be linked to food availability or ontogeny. The response to CO_2_ exposure is, however, consistent amongst seasons for shell condition and, to a lesser degree, a subset of the transcripts. This suggests that there is specificity and repeatability in these metrics, making them viable tools for bioindicator implementation. The low-cost, low-tech design of the shell condition workflow, as well as the fully developed intensity and duration characterization of the shell condition response, makes it an attractive choice for immediate implementation into biomonitoring and modelling efforts over the previously proposed pteropod bioindicator metrics (Bednaršek *et al.,* 2017). Although prior work seems to indicate that the 1.5 aragonite saturation state threshold holds for multiple species of *Limacina* (Bednarsek *et al.,* 2019), the quantitative relationships between saturation state and transparency should be explored in other species to determine the broader applicability of these equations.

## Supplementary Material

Web_Material_coae040

## Data Availability

All metabolic, shell condition and carbonate chemistry data are available at BCO-DMO (https://www.bco-dmo.org/project/2263). Gene expression data is available at Genbank (NCBI BioProject PRJNA260534).

## References

[ref1] Bates NR , Astor YM, Church MJ, Currie K, Dore JE, González-Dávila M, Lorenzoni L, Muller-Karger F, Olafsson J, Santana-Casiano JM (2014) A time-series view of changing surface ocean chemistry due to ocean uptake of anthropogenic co_2_. Oceanography 27: 126–141. 10.5670/oceanog.2014.16.

[ref2] Baumann H , Cross EL, Murray CS (2018) Robust quantification of fish early life co_2_ sensitivities via serial experimentation. Biol Lett 14: 20180408. 10.1098/rsbl.2018.0408. 30487256 PMC6283935

[ref3] Bé AWH , Gilmer RW (1977) A zoogeographic and taxonomic review of euthecosomatous pteropoda. In A Ramsay, ed, Oceanic Micropalaeontology Vol. 1. Academic Press, London, pp. 733–808.

[ref4] Bednaršek N , Carter BR, McCabe RM, Feely RA, Howard E, Chavez FP, Elliott M, Fisher JL, Jahncke J, Siegrist Z (2022) Pelagic calcifiers face increased mortality and habitat loss with warming and ocean acidification. Ecol Appl 32: e2674. 10.1002/eap.2674. 35584131 PMC9786838

[ref5] Bednarsek N , Feely RA, Howes EL, Hunt B, Kessouri F, León P, Lischka S, Maas AE, McLaughlin K, Nezlin N (2019) Systematic review and meta-analysis towards synthesis of thresholds of ocean acidification impacts on calcifying pteropods and interactions with warming. Front Mar Sci 6: 227. 10.3389/fmars.2019.00227.

[ref6] Bednaršek N , Feely RA, Pelletier G, Desmet F (2023) Global synthesis of the status and trends of ocean acidification impacts on shelled pteropods. Oceanography 36: 130–137. 10.5670/oceanog.2023.210.

[ref7] Bednarsek N , Johnson J, Feely R (2016) Comment on peck et al: vulnerability of pteropod (*Limacina helicina*) to ocean acidification: shell dissolution occurs despite an intact organic layer. Deep-Sea Res II Top Stud Oceanogr 127: 53–56. 10.1016/j.dsr2.2016.03.006.

[ref8] Bednaršek N , Klinger T, Harvey CJ, Weisberg S, McCabe RM, Feely RA, Newton J, Tolimieri N (2017) New ocean, new needs: application of pteropod shell dissolution as a biological indicator for marine resource management. Ecol Indic 76: 240–244. 10.1016/j.ecolind.2017.01.025.

[ref9] Bednarsek N , Ohman M (2015) Changes in pteropod distributions and shell dissolution across a frontal system in the California current system. Mar Ecol Prog Ser 523: 93–103. 10.3354/meps11199.

[ref10] Bednaršek N , Tarling GA, Bakker DCE, Fielding S, Jones EM, Venables HJ, Ward P, Kuzirian A, Leze B, Feely RA (2012) Extensive dissolution of live pteropods in the Southern Ocean. Nat Geosci 5: 881–885. 10.1038/ngeo1635.

[ref11] Bergan AJ (2017) Pteropod Shell Condition, Locomotion, and Long-term Population Trends in the Context of Ocean Acidification and Environmental Change. Massachusetts Institute of Technology, Cambridge, MA.

[ref12] Bergan AJ , Lawson GL, Maas AE, Wang ZA (2017) The effect of elevated carbon dioxide on the sinking and swimming of the shelled pteropod *Limacina retroversa*. ICES Journal of Marine Science 74: 1893–1905. 10.1093/icesjms/fsx008.

[ref13] Bolger AM , Lohse M, Usadel B (2014) Trimmomatic: a flexible trimmer for illumina sequence data. Bioinformatics 30: 2114–2120. 10.1093/bioinformatics/btu170. 24695404 PMC4103590

[ref14] Brown NE , Bernhardt JR, Anderson KM, Harley CD (2018) Increased food supply mitigates ocean acidification effects on calcification but exacerbates effects on growth. Sci Rep 8: 1–9. 29955096 10.1038/s41598-018-28012-w PMC6023940

[ref15] Comeau S , Jeffree R, Teyssié JL, Gattuso JP (2010) Response of the arctic pteropod *Limacina helicina* to projected future environmental conditions. PloS One 5: e11362. 10.1371/journal.pone.0011362. 20613868 PMC2894046

[ref16] Cominassi L , Moyano M, Claireaux G, Howald S, Mark FC, Zambonino-Infante J-L, Peck MA (2020) Food availability modulates the combined effects of ocean acidification and warming on fish growth. Sci Rep 10: 2338–2312. 10.1038/s41598-020-58846-2. 32047178 PMC7012865

[ref17] Cooley SR , Rheuban JE, Hart DR, Luu V, Glover DM, Hare JA, Doney SC (2015) An integrated assessment model for helping the United States sea scallop (*Placopecten magellanicus*) fishery plan ahead for ocean acidification and warming. PloS One 10: e0124145. 10.1371/journal.pone.0124145. 25945497 PMC4422659

[ref18] Cross JN , Turner JA, Cooley SR, Newton JA, Azetsu-Scott K, Chambers RC, Dugan D, Goldsmith K, Gurney-Smith H, Harper AR et al. (2019) Building the knowledge-to-action pipeline in North America: connecting ocean acidification research and actionable decision support. Front Mar Sci 6: 356. 10.3389/fmars.2019.00356.

[ref19] Dickson AG (1990) Thermodynamics of the dissociation of boric acid in synthetic seawater from 273.15 to 318.15 k. Deep Sea Research Part A Oceanographic Research Papers 37 37: 755–766. 10.1016/0198-0149(90)90004-F.

[ref20] Dickson AG , Sabine CL, Christian JR (2007) Guide to best practices for ocean CO_2_ measurements. PICES Special Publication 3, p. 191

[ref21] Doney SC , Busch DS, Cooley SR, Kroeker KJ (2020) The impacts of ocean acidification on marine ecosystems and reliant human communities. Annu Rev Env Resour 45: 83–112. 10.1146/annurev-environ-012320-083019.

[ref22] Doney SC , Fabry VJ, Feely RA, Kleypas JA (2009) Ocean acidification: the other co_2_ problem. Ann Rev Mar Sci 1: 169–192. 10.1146/annurev.marine.010908.163834. 21141034

[ref23] Doo SS , Kealoha A, Andersson A, Cohen AL, Hicks TL, Johnson ZI, Long MH, McElhany P, Mollica N, Shamberger KE et al. (2020) The challenges of detecting and attributing ocean acidification impacts on marine ecosystems. ICES Journal of Marine Science 77: 2411–2422. 10.1093/icesjms/fsaa094.

[ref24] Espinel-Velasco N , Hoffmann L, Agüera A, Byrne M, Dupont S, Uthicke S, Webster NS, Lamare M (2018) Effects of ocean acidification on the settlement and metamorphosis of marine invertebrate and fish larvae: a review. Mar Ecol Prog Ser 606: 237–257. 10.3354/meps12754.

[ref25] Friedlingstein P , O'Sullivan M, Jones MW, Andrew RM, Gregor L, Hauck J, Le Quéré C, Luijkx IT, Olsen A, Peters GP et al. (2022) Global carbon budget 2022. Earth Syst Sci Data 14: 4811–4900. 10.5194/essd-14-4811-2022.

[ref26] Gruber N , Clement D, Carter BR, Feely RA, Van Heuven S, Hoppema M, Ishii M, Key RM, Kozyr A, Lauvset SK (2019) The oceanic sink for anthropogenic co_2_ from 1994 to 2007. Science 363: 1193–1199. 10.1126/science.aau5153. 30872519

[ref27] Haas BJ , Papanicolaou A, Yassour M, Grabherr M, Blood PD, Bowden J, Couger MB, Eccles D, Li B, Lieber M et al. (2013) De novo transcript sequence reconstruction from rna-seq using the trinity platform for reference generation and analysis. Nat Protoc 8: 1494–1512. 10.1038/nprot.2013.084. 23845962 PMC3875132

[ref28] Hancock AM , King CK, Stark JS, McMinn A, Davidson AT (2020) Effects of ocean acidification on Antarctic marine organisms: a meta-analysis. Ecol Evol 10: 4495–4514. 10.1002/ece3.6205. 32489613 PMC7246202

[ref29] Hare JA , Morrison WE, Nelson MW, Stachura MM, Teeters EJ, Griffis RB, Alexander MA, Scott JD, Alade L, Bell RJ et al. (2016) A vulnerability assessment of fish and invertebrates to climate change on the northeast US continental shelf. PloS One 11: e0146756. 10.1371/journal.pone.0146756. 26839967 PMC4739546

[ref30] Howes EL , Bednaršek N, Büdenbender J, Comeau S, Doubleday A, Gallager SM, Hopcroft RR, Lischka S, Maas AE, Bijma J et al. (2014) Sink and swim: a status review of thecosome pteropod culture techniques. J Plankton Res 36: 299–315. 10.1093/plankt/fbu002.

[ref31] Ikeda T (2014) Metabolism and chemical composition of marine pelagic gastropod molluscs: a synthesis. J Oceanogr 70: 289–305. 10.1007/s10872-014-0231-y.

[ref32] IPCC (2022) Climate Change 2022: Impacts, Adaptation, and Vulnerability. Contribution of Working Group II to the Sixth Assessment Report of the Intergovernmental Panel on Climate Change. Cambridge University Press, Cambridge, UK and New York, NY, USA, p. e0146756.

[ref33] Johnson KM , Hofmann GE (2017) Transcriptomic response of the Antarctic pteropod Limacina helicina Antarctica to ocean acidification. BMC Genomics 18: 812. 10.1186/s12864-017-4161-0. 29061120 PMC5653985

[ref34] Koh H , Lee J, Han S, Park H, Shin S, Lee S (2015) A transcriptomic analysis of the response of the Arctic pteropod *Limacina helicina* to carbon dioxide-driven seawater acidification. Polar Biology 38: 1727–1740. 10.1007/s00300-015-1738-4.

[ref35] Kroeker KJ , Kordas RL, Crim RN, Singh GG (2010) Meta-analysis reveals negative yet variable effects of ocean acidification on marine organisms. Ecol Lett 13: 1419–1434. 10.1111/j.1461-0248.2010.01518.x. 20958904

[ref36] Langmead B , Salzberg SL (2012) Fast gapped-read alignment with bowtie 2. Nat Methods 9: 357–359. 10.1038/nmeth.1923. 22388286 PMC3322381

[ref37] Lee K , Kim T-W, Byrne RH, Millero FJ, Feely RA, Liu Y-M (2010) The universal ratio of boron to chlorinity for the North Pacific and North Atlantic oceans. Geochim Cosmochim Acta 74: 1801–1811. 10.1016/j.gca.2009.12.027.

[ref38] León P , Bednaršek N, Walsham P, Cook K, Hartman SE, Wall-Palmer D, Hindson J, Mackenzie K, Webster L, Bresnan E (2020) Relationship between shell integrity of pelagic gastropods and carbonate chemistry parameters at a Scottish coastal observatory monitoring site. ICES Journal of Marine Science 77: 436–450.

[ref39] Leung JY , Zhang S, Connell SD (2022) Is ocean acidification really a threat to marine calcifiers? A systematic review and meta-analysis of 980+ studies spanning two decades. Small 18: e2107407. 10.1002/smll.202107407. 35934837

[ref40] Li B , Dewey CN (2011) Rsem: accurate transcript quantification from rna-seq data with or without a reference genome. BMC Bioinformatics 12: 323. 10.1186/1471-2105-12-323. 21816040 PMC3163565

[ref41] Lischka S , Riebesell U (2012) Synergistic effects of ocean acidification and warming on overwintering pteropods in the Arctic. Glob Chang Biol 18: 3517–3528. 10.1111/gcb.12020.

[ref42] Lueker TJ , Dickson AG, Keeling CD (2000) Ocean pco_2_ calculated from dissolved inorganic carbon, alkalinity, and equations for k1 and k2: validation based on laboratory measurements of co_2_ in gas and seawater at equilibrium. Mar Chem 70: 105–119. 10.1016/S0304-4203(00)00022-0.

[ref43] Ma E , Haddad GG (1997) Anoxia regulates gene expression in the central nervous system of *Drosophila melanogaster*. Mol Brain Res 46: 325–328. 10.1016/S0169-328X(97)00074-0. 9191110

[ref44] Maas AE , Elder LE, Dierssen HM, Seibel BA (2011) Metabolic response of Antarctic pteropods (mollusca: Gastropoda) to food deprivation and regional productivity. Mar Ecol Prog Ser 441: 129–139. 10.3354/meps09358.

[ref45] Maas AE , Lawson GL, Bergan AJ, Tarrant AM (2018) Exposure to co_2_ influences metabolism, calcification, and gene expression of the thecosome pteropod *Limacina retroversa*. J Exp Biol 221: 164400. 10.1242/jeb.164400 29191863

[ref46] Maas AE , Lawson GL, Bergan AJ, Wang ZA, Tarrant AM (2020) Seasonal variation in physiology and shell condition of the pteropod Limacina retroversa in the Gulf of Maine relative to life cycle and carbonate chemistry. Prog Oceanogr 186: 102371. 10.1016/j.pocean.2020.102371.

[ref47] Maas AE , Lawson GL, Tarrant AM (2015) Transcriptome-wide analysis of the response of the thecosome pteropod Clio pyramidata to short-term co2 exposure. Comparative Biochemistry and Physiology Part D: Genomics and Proteomics 16: 1–9. 10.1016/j.cbd.2015.06.002. 26143042

[ref48] Mahadav A , Kontsedalov S, Czosnek H, Ghanim M (2009) Thermotolerance and gene expression following heat stress in the whitefly *Bemisia tabaci* b and q biotypes. Insect Biochem Mol Biol 39: 668–676. 10.1016/j.ibmb.2009.08.002. 19683053

[ref49] Manno C , Bednaršek N, Tarling GA, Peck VL, Comeau S, Adhikari D, Bakker DCE, Bauerfeind E, Bergan AJ, Berning MI et al. (2017) Shelled pteropods in peril: assessing vulnerability in a high co_2_ ocean. Biol Rev 169: 132–145.

[ref50] McLaughlin K , Weisberg SB, Dickson AG, Hofmann GE, Newton JA, Aseltine-Neilson D, Barton A, Cudd S, Feely RA, Jefferds IW (2015) Core principles of the California current acidification network: linking chemistry, physics, and ecological effects. Oceanography 25: 160–169. 10.5670/oceanog.2015.39.

[ref51] Mekkes L , Renema W, Bednaršek N, Alin SR, Feely RA, Huisman J, Roessingh P, Peijnenburg KT (2021) Pteropods make thinner shells in the upwelling region of the California current ecosystem. Sci Rep 11: 1–11. 10.1038/s41598-021-81131-9. 33462349 PMC7814018

[ref52] Miller M , Oakes R, Covert P, Ianson D, Dower J (2023) Evidence for an effective defence against ocean acidification in the key bioindicator pteropod Limacina helicina. ICES Journal of Marine Science 80: 1329–1341. 10.1093/icesjms/fsad059.

[ref53] Moya A , Howes EL, Lacoue-Labarthe T, Forêt S, Hanna B, Medina M, Munday PL, Ong JS, Teyssié JL, Torda G et al. (2016) Near future pH conditions severely impact calcification, metabolism and the nervous system in the pteropod *Heliconoides inflatus*. Glob Chang Biol 22: 3888–3900. 10.1111/gcb.13350. 27279327

[ref54] Moya A , Huisman L, Ball E, Hayward D, Grasso L, Chua C, Woo H, Gattuso JP, Forêt S, Miller D (2012) Whole transcriptome analysis of the coral A*cropora millepora* reveals complex responses to co_2_ driven acidification during the initiation of calcification. Mol Ecol 21: 2440–2454. 10.1111/j.1365-294X.2012.05554.x. 22490231

[ref55] Niemi A , Bednaršek N, Michel C, Feely RA, Williams W, Azetsu-Scott K, Walkusz W, Reist JD (2021) Biological impact of ocean acidification in the Canadian Arctic: widespread severe pteropod shell dissolution in Amundsen Gulf. Front Mar Sci 8: 222. 10.3389/fmars.2021.600184.

[ref56] Oakes RL , Peck VL, Manno C, Bralower TJ (2019) Impact of preservation techniques on pteropod shell condition. Polar Biology 42: 257–269. 10.1007/s00300-018-2419-x.

[ref57] O'Donnell MJ , Todgham AE, Sewell MA, Hammond LTM, Ruggiero K, Fangue NA, Zippay ML, Hofmann GE (2010) Ocean acidification alters skeletogenesis and gene expression in larval sea urchins. Mar Ecol Prog Ser 398: 157–171. 10.3354/meps08346.

[ref58] Pansch C , Schaub I, Havenhand J, Wahl M (2014) Habitat traits and food availability determine the response of marine invertebrates to ocean acidification. Glob Chang Biol 20: 765–777. 10.1111/gcb.12478. 24273082

[ref59] Peck VL , Tarling GA, Manno C, Harper EM et al. (2016a) Reply to comment by Bednarsek. Deep-Sea Res II Top Stud Oceanogr 127: 57–59. 10.1016/j.dsr2.2016.03.007.

[ref60] Peck VL , Tarling GA, Manno C, Harper EM, Tynan E (2016b) Outer organic layer and internal repair mechanism protects pteropod *Limacina helicina* from ocean acidification. Deep-Sea Res II Top Stud Oceanogr 127: 41–52. 10.1016/j.dsr2.2015.12.005.

[ref61] Pierrot D , Lewis E, Wallace D (2006) CO2SYS DOS program developed for CO2 system calculations. In Carbon Dioxide Information Analysis Center. Oak Ridge National Laboratory, US Department of Energy, Oak Ridge, TN. ORNL/CDIAC-105.

[ref62] Ramos-Silva P , Odendaal M-L, Wall-Palmer D, Mekkes L, Peijnenburg KT (2022) Transcriptomic responses of adult versus juvenile atlantids to ocean acidification. Front Mar Sci 9: 801458. 10.3389/fmars.2022.801458.

[ref63] Riebesell U , Fabry VJ, Hansson L, Gattuso JP (2010) Guide to Best Practices for Ocean Acidification Research and Data Reporting. Publications Office of the European Union, Luxembourg.

[ref64] Robinson MD , McCarthy DJ, Smyth GK (2010) Edger: a bioconductor package for differential expression analysis of digital gene expression data. Bioinformatics 26: 139–140. 10.1093/bioinformatics/btp616. 19910308 PMC2796818

[ref65] Seibel BA , Dymowska A, Rosenthal J (2007) Metabolic temperature compensation and co-evolution of locomotory performance in pteropod moluscs. Integr Comp Biol 47: 880–891. 10.1093/icb/icm089. 21669767

[ref66] Seibel BA , Maas AE, Dierssen HM (2012) Energetic plasticity underlies a variable response to ocean acidification in the pteropod, *Limacina helicina Antarctica*. PloS One 7: e30464. 10.1371/journal.pone.0030464. 22536312 PMC3335044

[ref67] Simão FA , Waterhouse RM, Ioannidis P, Kriventseva EV, Zdobnov EM (2015) Busco: assessing genome assembly and annotation completeness with single-copy orthologs. Bioinformatics 31: 3210–3212. 10.1093/bioinformatics/btv351. 26059717

[ref68] Snyder JT , Murray CS, Baumann H (2018) Potential for maternal effects on offspring co_2_ sensitivities in the Atlantic silverside (*Menidia menidia*). J Exp Mar Biol Ecol 499: 1–8. 10.1016/j.jembe.2017.11.002.

[ref69] Thabet AA , Maas AE, Lawson GL, Tarrant AM (2015) Life cycle and early development of the thecosomatous pteropod *Limacina retroversa* in the Gulf of Maine, including the effect of elevated co_2_ levels. Mar Biol 162: 2235–2249. 10.1007/s00227-015-2754-1.

[ref70] Thomsen J , Casties I, Pansch C, Körtzinger A, Melzner F (2013) Food availability outweighs ocean acidification effects in juvenile *Mytilus edulis*: laboratory and field experiments. Glob Chang Biol 19: 1017–1027. 10.1111/gcb.12109. 23504880

[ref71] Tilbrook B , Jewett EB, DeGrandpre MD, Hernandez-Ayon JM, Feely RA, Gledhill DK, Hansson L, Isensee K, Kurz ML, Newton JA et al. (2019) An enhanced ocean acidification observing network: from people to technology to data synthesis and information exchange. Front Mar Sci 6: 337. 10.3389/fmars.2019.00337.

[ref72] Todgham AE , Hofmann GE (2009) Transcriptomic response of sea urchin larvae *Strongylocentrotus purpuratus* to co_2_-driven seawater acidification. J Exp Biol 212: 2579–2594. 10.1242/jeb.032540. 19648403

[ref73] Wang ZA , Lawson GL, Pilskaln CH, Maas AE (2017) Seasonal controls of aragonite saturation states in the Gulf of Maine. J Geophys Res Oceans 122: 372–389. 10.1002/2016JC012373.

[ref74] Wang ZA , Wanninkhof R, Cai WJ, Byrne RH, Hu X, Peng TH, Huang WJ (2013) The marine inorganic carbon system along the Gulf of Mexico and Atlantic coasts of the United States: insights from a transregional coastal carbon study. Limnol Oceanogr 58: 325–342. 10.4319/lo.2013.58.1.0325.

[ref75] Weisberg SB , Bednaršek N, Feely RA, Chan F, Boehm AB, Sutula M, Ruesink JL, Hales B, Largier JL, Newton JA (2016) Water quality criteria for an acidifying ocean: challenges and opportunities for improvement. Ocean & Coastal Management 126: 31–41. 10.1016/j.ocecoaman.2016.03.010.

[ref76] White MM , McCorkle DC, Mullineaux LS, Cohen AL (2013) Early exposure of bay scallops (*Argopecten irradians*) to high co_2_ causes a decrease in larval shell growth. PloS One 8: e61065. 10.1371/journal.pone.0061065. 23596514 PMC3626597

[ref77] Woo S , Denis V, Won H, Shin K, Lee G, Lee T-K, Yum S (2013) Expressions of oxidative stress-related genes and antioxidant enzyme activities in *Mytilus galloprovincialis* (bivalvia, mollusca) exposed to hypoxia. Zoological Studies 52: 1–8. 10.1186/1810-522X-52-15.

