# Supplementary material for: Sea butterflies in a pickle: reliable biomarkers and seasonal sensitivity of Limacina retroversa to ocean acidification in the Gulf of Maine
Source: Conserv Physiol. 2024 Jun 21;12(1):coae040. doi: 10.1093/conphys/coae040 (PMC11194183; doi:10.1093/conphys/coae040)
Supplement: Web_Material_coae040 [file web_material_coae040.zip › Maas_pteropod_biomarkers_supplement_R1_(7)_coae040.pdf]

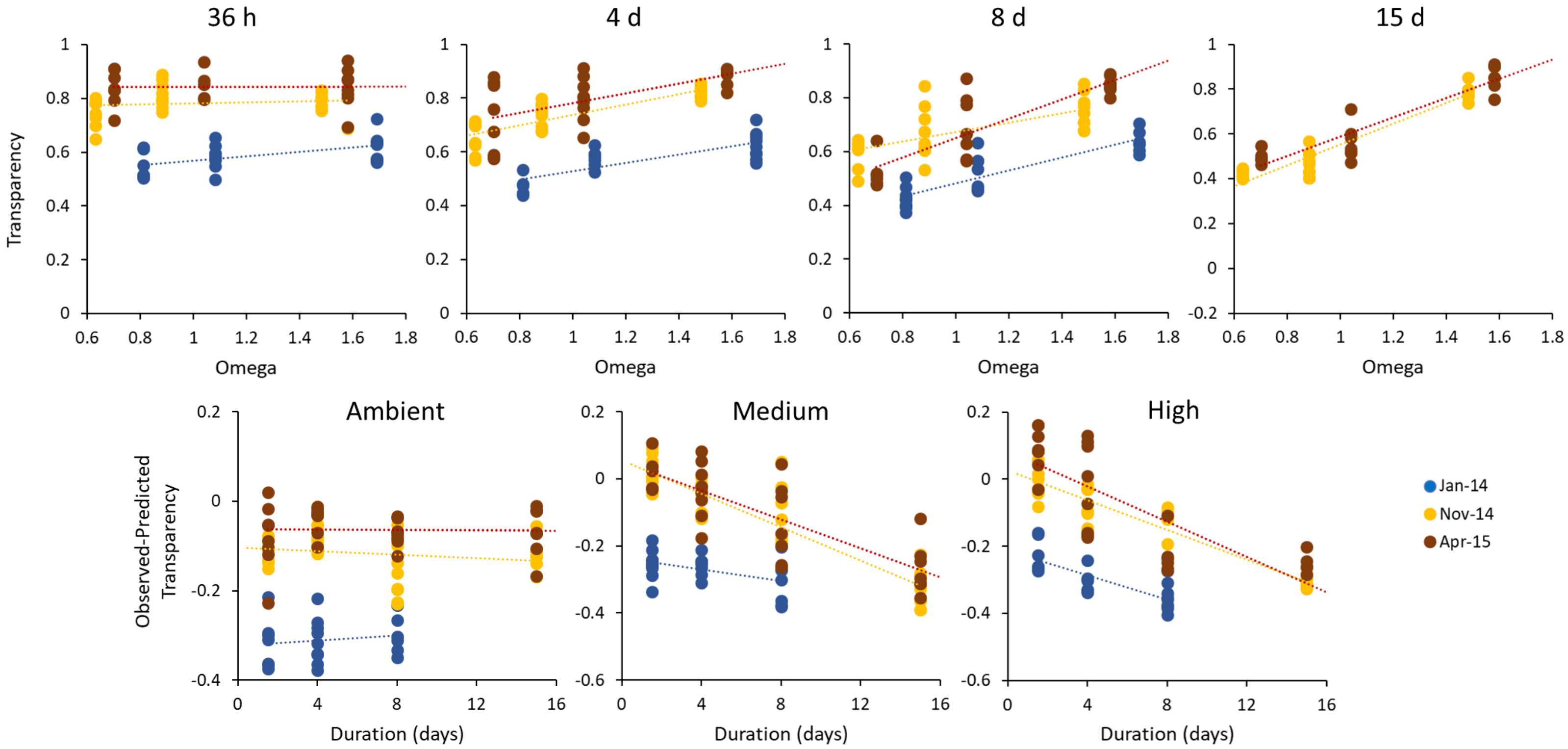

Figure S1: Detailed seasonal patterns of exposure duration effect on shell transparency. *There was typically a consistent effect of the intensity of* CO_2 *exposure (aragonite saturation state) on shell transparency during each day of the duration experiments (upper row), with the sampling period (color) having minimal effect on the slope of the relationship (power regression). Analysis of the duration effect (lower row), analyzed by subtracting the observed transparency from the calculated predicted transparency (based on the experimental saturation state and equation 2), demonstrated no interactive effect between duration and sampling period (color), and suggests that there was no effect of duration on the ambient treatments.*

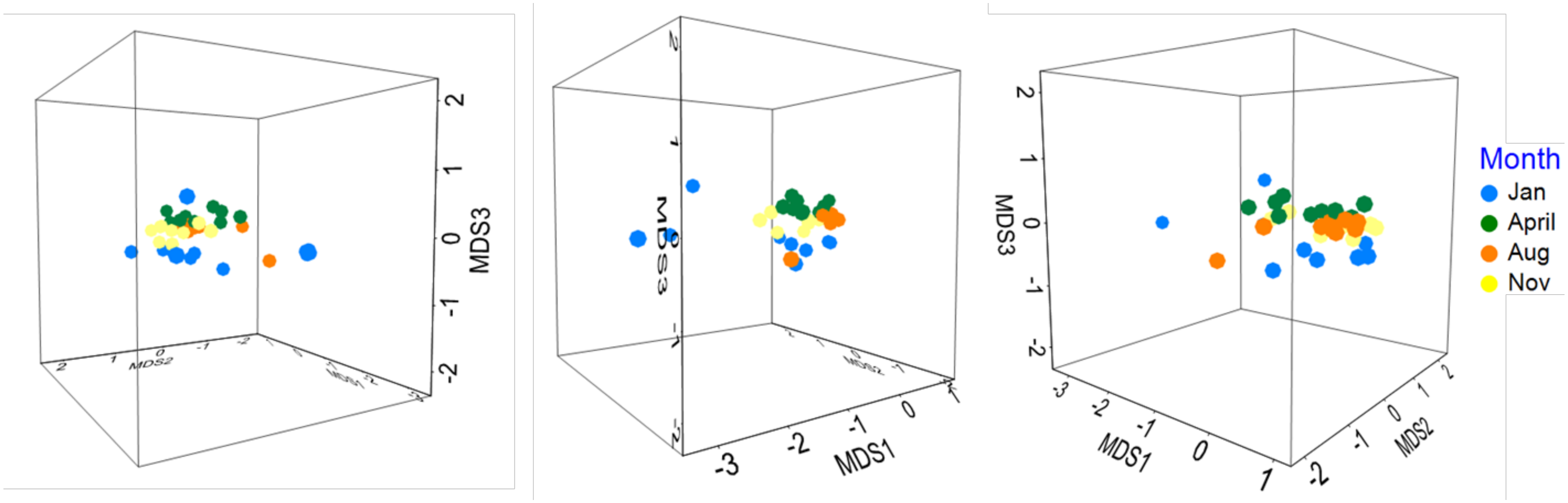

Figure S2: nMDS of Differential Gene Expression 3D. *Three rotated perspectives of the third dimension of the DE nMDS plot (visualized in Fig. 4B) demonstrates the seasonal clumping of the data that is less obvious in the 2D version.*
